# A CRHR1 antagonist prevents synaptic loss and memory deficits in a trauma-induced delirium-like syndrome

**DOI:** 10.1038/s41380-020-0659-y

**Published:** 2020-02-12

**Authors:** Silvia Cursano, Chiara R. Battaglia, Carolina Urrutia-Ruiz, Stefanie Grabrucker, Michael Schön, Jürgen Bockmann, Sonja Braumüller, Peter Radermacher, Francesco Roselli, Markus Huber-Lang, Tobias M. Boeckers

**Affiliations:** 1 Institute for Anatomy and Cell Biology, Ulm University, Albert-Einstein-Allee 11, 89081 Ulm, Germany; 2 International Graduate School in Molecular Medicine, IGradU, 89081 Ulm, Germany; 3 Department of Biological Sciences, University of Limerick, Limerick, V94 PH61 Ireland; 4 Institute for Anesthesiological Pathophysiology, Ulm University, Helmholtzstr. 8/1, 89081 Ulm, Germany; 5 Clinic for Neurology, Ulm University, 89081 Ulm, Germany; 6 Institute of Clinical and Experimental Trauma-Immunology, Ulm University, 89081 Ulm, Germany

**Keywords:** Depression, Neuroscience, Psychology

## Abstract

Older patients with severe physical trauma are at high risk of developing neuropsychiatric syndromes with global impairment of cognition, attention, and consciousness. We employed a thoracic trauma (TxT) mouse model and thoroughly analyzed age-dependent spatial and temporal posttraumatic alterations in the central nervous system. Up to 5 days after trauma, we observed a transient 50% decrease in the number of excitatory synapses specifically in hippocampal pyramidal neurons accompanied by alterations in attention and motor activity and disruption of contextual memory consolidation. In parallel, hippocampal corticotropin-releasing hormone (CRH) expression was highly upregulated, and brain-derived neurotrophic factor (BDNF) levels were significantly reduced. In vitro experiments revealed that CRH application induced neuronal autophagy with rapid lysosomal degradation of BDNF via the NF-κB pathway. The subsequent synaptic loss was rescued by BDNF as well as by specific NF-κB and CRH receptor 1 (CRHR1) antagonists. In vivo, the chronic application of a CRHR1 antagonist after TxT resulted in reversal of the observed histological, molecular, and behavioral alterations. The data suggest that neuropsychiatric syndromes (i.e., delirium) after peripheral trauma might be at least in part due to the activation of the hippocampal CRH/NF-κB/BDNF pathway, which results in a dramatic loss of synaptic contacts. The successful rescue by stress hormone receptor antagonists should encourage clinical trials focusing on trauma-induced delirium and/or other posttraumatic syndromes.

## Introduction

The abrupt decline of mental function (termed delirium) is a common neuropsychiatric syndrome, with a prevalence of ~20% in the general hospital setting [1, 2]. In comparison with control patients, patients with delirium have a poorer prognosis and experience an increased length of hospital stay [3], and delirium has been recognized as an important cause of the growing financial burden in an aging society. Delirium is characterized by a general disorientation accompanied by cognitive impairment, changes in arousal and in some cases hallucinations and delusions [4, 5]. The course of the illness is typically short, lasting only a few days to weeks, and delirium is usually reversible [6]. In addition to physical trauma, common causes of delirium include substance intoxication or withdrawal, hyperglycemia, hyperthermia, infections, and brain lesions [7, 8]. Interestingly, advanced age is one of the most predictive risk factors for delirium [9], and following physical trauma, a higher multiple organ failure score favors the occurrence of postinjury delirium [7].

Because of the etiological complexity of delirium, the unique disease course in different patients and the problem of defining and operationalizing the syndrome, treatment options, and interventions are limited [10]. Despite its clinical relevance, basic research on trauma-related neuropsychiatric syndromes is complex, and mouse models must be carefully evaluated according to the different types of validity [11]. Therefore, our understanding of the pathophysiology that induces neuropsychiatric alterations after trauma is sparse, and our knowledge on periphery–brain interactions and communication after traumatic events is limited. On the other hand, there have been important studies on the effect of physical stress on the central nervous system (CNS) and the role of stress hormones (especially corticotropin-releasing hormone, CRH) in mediating morphological alterations of hippocampal spines and synapses via CRH receptor 1 (CRHR1) activation [12–14]. The authors introduce a CRH/calpain/RhoA-dependent mechanism that mediates actin polymerization and depolymerization at synaptic sites. Other lines of evidence support the importance of neurotrophic factors (especially brain-derived neurotrophic factor, BDNF) [15, 16] in modulating synaptic and spine plasticity after peripheral stress [17] and/or alterations in stress hormones [18]. Recently, the hypothesis that delirium and hospital-acquired weakness are caused by synaptic dysfunctions was proposed [19].

To elucidate some of the issues discussed above, we employed a peripheral trauma model and searched for morphological and molecular neurobiological correlates/pathways of posttraumatic disturbances in the brain. To that end, we made use of a blunt chest trauma (TxT) mouse model that presents complex injury of the lung in combination with partial damage to connective and supporting tissue [20]. We screened for structural and molecular alterations in the CNS, especially at the synaptic level, and compared the impact of trauma on young and aged mice at different posttraumatic time intervals.

In this study, we found that ~50% of pyramidal neuron excitatory synapses within the hippocampal formation were lost, attention and motor activity were altered, and contextual memory in a fear conditioning paradigm was greatly reduced 5 days after trauma in aged animals. Interestingly, the amount of CRH released from hippocampal interneurons was increased, while mature BDNF levels were almost undetectable. Next, we identified and characterized the neuronal CRH-CRHR1-NF-κB pathway, which induces autophagy and rapid lysosomal degradation of BDNF, to explain local transitional synaptic loss after peripheral trauma. Finally, we rescued TxT-induced synaptic loss and behavioral deficits by chronic treatment with a CRHR1 antagonist. Since we used compounds that are already in medical use, our findings should initiate clinical studies that concentrate on central modulators of stress for the treatment of delirium and other trauma-related insults.

## Materials and methods

### Animals

Male C57BL/6JRj mice (8–10 weeks old (body weight 25 ± 1.5 g) and 22–24 months old (body weight 30 ± 1.5 g)) were group-housed on a 12/12-h light/dark schedule (lights on at 7:0 A.M.) with ad libitum access to food and water before and after blast exposure. All animal experiments were performed in compliance with the guidelines for the welfare of experimental animals issued by the Federal Government of Germany and approved by the Regierungspraesidium Tuebingen and the local ethics committee at Ulm University; ID number: 1233.

### Thoracic trauma

Thoracic trauma (TxT) was always induced during the morning. Mice were anesthetized with a mixture 2.5% sevoflurane (Sevorane™, Abbott, Wiesbaden, Germany) and 97.5% oxygen at a continuous flow of 0.5 l min^−1^ and a FiO2 of 1.0. The mice were fixed to an acrylic glass plate in the supine position, and the abdomen and chest were shaved. Before termination of anesthesia, buprenorphine (0.03 mg kg^−1^ body weight) was injected subcutaneously to provide suitable analgesia. TxT was induced by a single blast wave centered on the thorax as previously described by Ehrnthaller et al. [20]. One group was used as sham controls, and these animals were subjected to the same experimental conditions, but no blast wave was delivered. Analysis was performed 6 h (for a few experiments), 5 days, 10 days, and 18 days post injury (dpi); at these time points, we collected the brains and blood to perform subsequent analyses. To evaluate the general conditions and estimate the degree of distress, mice were observed every 2 h after trauma in the operating room, twice a day for the following week and once a day until the end of the experiment.

### Golgi staining procedures

Mice were sacrificed with an overdose of sevoflurane. The brains were removed and prepared for Golgi–Cox staining (FD Rapid GolgiStain^TM^ Kit). They were then cut sagittally into 150-µm-thick sections using a vibratome (Microm HM 650) and mounted on gelatin-coated slides.

### Imaging and quantification of dendritic spines

For the analysis, 9–10 neurons (pyramidal neurons and interneurons) from the CA1 and CA3 hippocampal subregions were analyzed per experimental condition. The apical dendrites in the stratum radiatum/lacunosum were analyzed. Cell types were distinguished based on morphological differences; spines from pyramidal neurons with cell bodies in the pyramidal layer and interneurons with cell bodies in the stratum radiatum were analyzed. Golgi-stained dendritic branches and spines were documented using a BZ-9000 Fluorescence Microscope **(**KEYENCE Corp.). Image Z-stacks (0.4-µm focal steps over the entire thickness of each dendrite) were collected with a ×100/1.4 oil lens and reconstructed using ImageJ software. A total of 5–8 dendrites per animal and three animals per group were evaluated. Spine density was expressed as the number of spines per 10 µm of dendrite length.

### Quantitative real-time PCR

Isolation of total RNA from mouse brain regions or rat hippocampal cell culture was performed using the RNeasy Mini kit (Qiagen) as described by the manufacturer. Thermal cycling and fluorescence detection were performed using the Rotor-Gene-Q real-time PCR machine (model 2- Plex HRM) (Qiagen). Cycle threshold values were calculated by Rotor-Gene Q software (version 2.0.2). All qRT-PCR reactions were run in triplicate for each time point and condition. Real-time quantitative PCR was carried out using oligonucleotides to investigate the expression of CRH, CRHR1, CRHR2, BDNF, and TrkB (validated primer pairs, Quantitect Primer Assay, Qiagen).

### Primary antibodies

The primary antibodies used for immunocytochemistry are described below. Shank2 (1:500) (“ppI-SAM pabSA5192”) was previously characterized [21–23]. The following primary antibodies were purchased from commercial suppliers: Vglut1 (1:500, Synaptic Systems GmbH, #135304), Gephyrin (1:500, Synaptic Systems GmbH, #147003), Vgat (1:500, Synaptic Systems GmbH, #131011), Iba1 (1:250, Wako Chemical GmbH, #NCNP24), GFAP (1:500, Sigma-Aldrich, #G3893*)*, NeuN (1:1000, Millipore, #MAB377), C1q (1:1000, Abcam, #182451), CRH (1:1000, Abcam, #8901), p62 (1:500, Abcam, #56416), Map2 (1:500, EnCor Biotechnology Inc., #CPCA-MAP2), Synaptotagmin-1 (1:500, Synaptic Systems, #105311C5), ATG5 (Abcam, #AB108327), NF-κB p65 (1:500, Santa Cruz, #SC8008), phospho-NF-κB (Thr435) (1:500, Thermo Fisher Scientific, #PA5–37724), phospho-IκBα (Ser32) (1:500, Cell Signaling, #2859), CRHR1 (1:500, Everest Biotech, #EB08035), and ChAT (1:250, Synaptic System, #297015).

The following primary antibodies were used for western blotting: Shank2 (1:2000) (“ppI-SAM pabSA5192”), Synapsin 1/2 (1:1000, Synaptic Systems GmbH, #160003), Gephyrin (1:500, Synaptic Systems GmbH, #147003), Vgat (1:500, Synaptic Systems GmbH, #131011), BDNF (1:500, Abcam, #203573), C3b/iC3b/C3c (1:200, Hycultec GmbH, #HM1065), Calpain-1 (1:500, Abcam, #39170), Actin (1:250000, Sigma-Aldrich, #A2228), Beclin-1 (BCN-1) (1:500, Novus Biologicals, #NB500249), LC3 (1:1000, Cell Signaling, #4599), and Lamp-2 (1:500, Thermo Fisher Scientific, #PA1655).

### Secondary antibodies

The secondary antibodies used for immunocytochemistry were coupled to Alexa Fluor® 488, 568, or 647 (1:500, all from Life Technologies). The secondary antibodies used for western blotting were HRP-conjugated (1:1000 Dako, Glostrup, Denmark).

### Slice preparation and immunohistochemistry

Animals were anesthetized (25% ketamine and 5% xylazine solubilized in a NaCl solution) and perfused with 25 ml cooled phosphate buffer saline (PBS) and 50 ml 4% PFA. Then, the brains were treated as previously described by Heise et al. [24]. Images of immunostained sections were taken using an upright fluorescence microscope (Axioskop, Zeiss) and Axiovision software (Zeiss). For in vivo studies, a confocal microscope (Leica SP5 or Leica SPE confocal microscope with a ×40 or ×63 objective) was used. For the magnified images, Fiji ImageJ (National Institute of Health, USA) and Bitplane Imaris software were used.

### Immunocytochemical analysis

Qualitative immunocytochemical analysis was carried out on three male mice. At least three coronal slices per animal were stained with each antibody/antibody combination to assure the representativeness of the staining. The intensity of immunostaining and colocalization were then analyzed using Fiji ImageJ and Bitplane Imaris software.

### Western blotting

Mice were sacrificed with an overdose of sevoflurane. Subcellular fractionation of brain tissue was performed as previously described [25].

### Transmission electron microscopy

Mice were perfused with 20 ml of solution 1 (0.5% heparinized saline solution) followed by 50 ml of solution 2 (2% paraformaldehyde, 2.5% glutaraldehyde, and 1% saccharose in 0.1 cacodylate phosphate buffer, pH 7.4). After perfusion, the mouse brains were dissected and postfixed in solution 3 (2% glutaraldehyde and 1% saccharose in 0.1 cacodylate buffered saline) at 4 °C overnight. The regions of interest (CA1 and CA3) were dissected in 1-mm^2^ pieces using a stereomicroscope. Then, the specimens were washed in 0.1 M PBS, postfixed with 2% osmium tetroxide for 1 h and dehydrated in an ascending propanol series (30%, 50%, 70%, and 90%). In addition, uranyl acetate diluted in denatured ethanol was used to contrast the specimens for 30 min at 37 °C. The epon-embedded specimens were cut into 0.5-µm sections with an ultratome, and the semithin sections were stained with toluidine blue. The defined subregions were cropped from the epon-embedded pieces, cut into ultrathin sections (70–80 nm) and collected on 300 mesh copper grids.

For the hippocampal neurons, transmission electron microscopy (TEM) was performed as previously reported [26].

### Transmission electron microscopy analysis

All analyzed specimens were investigated on a Jeol JEM 1400 transmission electron microscope at 120 kV. A magnification of ×25,000 was chosen to study synapses and the density of postsynaptic densities (PSDs) within an image section. Generally, synapses were counted within an area of 35.8 µm^2^. A magnification of ×80,000 was further applied to measure the PSD length and thickness. Only artifact-free synapses with clearly identifiable PSDs and presynaptic and postsynaptic terminals were selected for analysis. ImageJ software was used to determine the length, thickness, and volume of the PSDs. For the analysis of the SV density, membrane-associated vesicles and presynaptic terminal size were manually traced in ImageJ. Multivesicular bodies (MVBs) were identified as round-shaped vesicles surrounded by a single membrane that enclosed a variable number of small spherical vesicles within a matrix, according to the published criteria [27].

### Culturing and immunolabeling of rat hippocampal/cortical neurons for conventional fluorescence imaging

Primary rat embryo hippocampal/cortical cell culture and immunolabeling procedures were previously described by Schoen et al. [28].

### Image acquisition and analysis of rat hippocampal neurons

Images were obtained with an upright fluorescence microscope (Zeiss Axioskop 2 and Zeiss Imager Z1 with an apotome, Zeiss, Germany). Pictures were taken with Axiovison 4.7.1 software (Zeiss, Germany). Three different dendrites from three different neurons from three different wells were analyzed for each condition using Bitplane Imaris software.

### Experimental and pharmacological design

The CRH peptide (Bachem #H-2435) was maintained at a stock solution of 100 µM in sterile water, and then diluted to the desired concentration in NBM plus B27 just before use. Dissociated neurons on glass coverslips were incubated in 6- or 24-well plates at 36 °C for 30 min. The selective CRH receptor blocker NBI30775 (Hycultec GmbH, #HY-14127) and a selective GR blocker (Sigma-Aldrich, #RU38486) were dissolved in sterile DMSO, sonicated and used at a final concentration of 100 nM. BDNF (Peprotech #450–02) was prepared to a final concentration of 10 μg/ml. The blockers were applied alone for 5 min to allow the compound to bind the receptors. This was followed by application of a solution containing (1) NBI30775 and CRH or (2) NBI30775, RU38486, and CRH, and BDNF was immediately applied in combination with CRH for another 30 min.

### Phospho-protein array

Screening of 1318 phospho-proteins (Phospho Explorer Antibody Array #KAS02) was performed in hippocampal neurons under two experimental conditions: vehicle and 100 nM CRH. The detection of antibody arrays was performed in a fluorescence slide scanner (Genepix 4000B microarray scanner, Molecular Devices). The 16-bit images were analyzed using GenePixPro 6.1 software. For quantification, four replicates for each antibody and values from two independent experiments were used. The values were calculated relative to the average value of the corresponding control. Protein expression changes of >35% were considered relevant.

### Synaptotagmin assay

After 30 min of incubation with CRH with or without antagonist(s)/BDNF, synaptotagmin-1 (1:500) was administered for 30 min. Then, DIV14 neurons from all the experimental groups were fixed for ICC or rapidly processed for western blot analysis.

### NF-κB activation blockage

After 30 min of incubation with CRH, JSH23 (Abcam, #Ab144824) was added at a final concentration of 10 μM for 30 min to prevent NF-κB translocation to the nucleus. Then, neurons were fixed for ICC or rapidly processed for western blot analysis. To demonstrate the validity of the system, two other NF-κB activation blockers were tested: lactacystin (Abcam, #Ab141411) and SC-514, an IKKbeta inhibitor (Abcam, #ab144415).

### Lysosomal inhibition and reagents

DIV14 rat hippocampal neurons were treated with 100 nM CRH for 30 min in triplicate. Then, 50 μM leupeptin hemisulfate (Biomol, CAS #103476–89–7) and 5 μM E64 (Sigma-Aldrich, #E3132) were added for 30 min. Then, neurons from all the experimental groups were rapidly processed for western blot analysis.

### Behavioral test

Fear Conditioning System 46103 from Noldus/Ugo Basile was used to perform the trace cued and contextual test. The fear conditioning procedure was conducted over 3 days. During the first day (training day), the animals were placed in a unique context (25 × 17 × 17 box), and after 2 min of acclimation, they were exposed to a mild foot-shock (0.5 mA, 2 s) followed (20 s later) by a tone (85 dB, 2700 Hz, 20 s). The CS–US pairing was repeated five times. During the second day (auditory memory), the animals were placed in a different context, and after 2 min of acclimation, they were exposed three times to a tone with 2-min intervals (85 dB, 2700 Hz, 20 s). On the third day (contextual memory), the animals were exposed to the same context as that used for the training day for 8 min. On the fourth day, TxT was induced. Upon exposure to the same context or cue 5 or 18 days after TxT, animals exhibited a variety of fear responses, including freezing behavior [29, 30]. Freezing behavior was assessed as the percentage of time spent freezing (measured 1 min after the tone using Ethovision 12 software). Freezing was defined as a complete lack of movement, except for respiration [29, 30].

### In vivo antagonist administration

The selective CRH receptor blocker NBI30775 (Hycultec GmbH, #HY-14127) and a selective GR blocker (Sigma-Aldrich, #RU38486) were dissolved into sterile 70% PEG. NBI30775 (1 mg/kg) was administered subcutaneously immediately and 2, 6, and 10 h after TxT trauma and twice a day for 1 week. RU38486 (20 mg/kg) was administered subcutaneously immediately and 12 h after trauma and twice a day for 1 week. The TCCF test was performed as described above.

### Statistical analysis

The results are presented as the mean ± SEM. CRH/synapse-related immunostaining and all immunoblots were normalized to the value of the sham group (equal to 1) or, for the in vitro experiments, were normalized to the value of the vehicle group (equal to 1). One-way ANOVA followed by Bonferroni’s multiple comparison post hoc test was performed for most of the experiments to determine significant differences between experimental means. Some experiments were analyzed using two-tailed unpaired *T*-test or one-way ANOVA followed by the Dunnett post hoc test. The biological replicates from three mice were used for experiments with 2–3 technical replicates, except for the behavioral experiments, in which 6–8 mice were used. For the in vitro experiments, the biological triplicates were derived from 3–5 technical replicates. The 95% confidence interval was considered statistically significant. GraphPad Prism 7.0 was used to perform all statistical analyses.

## Results

### Thoracic trauma (TxT) leads to a selective loss of hippocampal excitatory synapses but no memory deficits in young mice

**Fig. 1:**
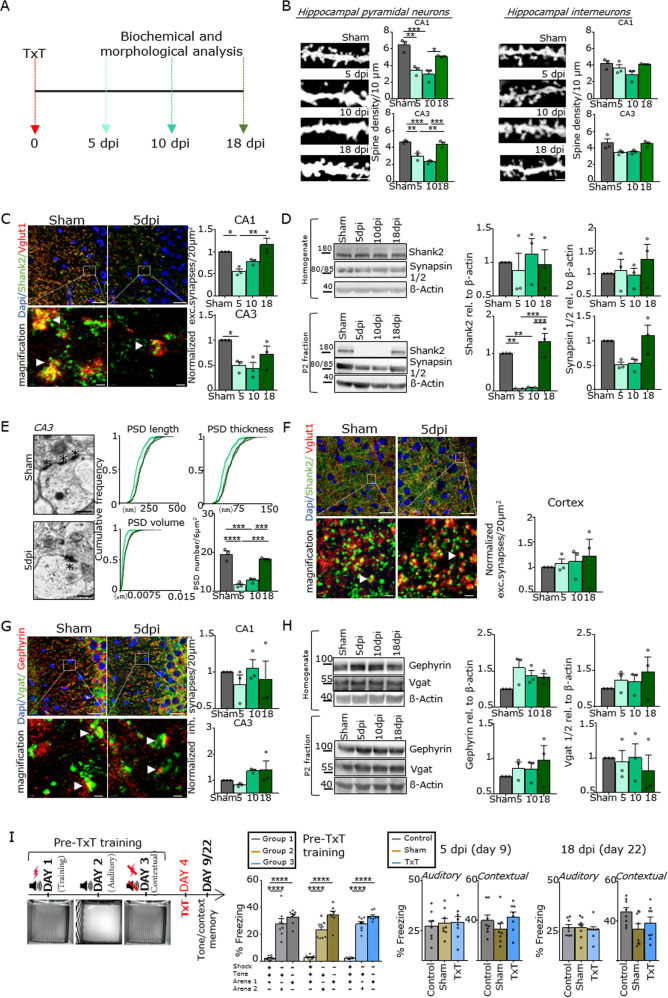
Loss of hippocampal excitatory synapses after TxT. **a** Schematic design of the experimental setup. **b** Analysis of spine density using Golgi staining in (left) pyramidal neurons and (right) interneurons (scale bar = 3 µm). **c** Immunohistochemical (IHC) staining and quantification of excitatory synapses in the hippocampus using the postsynaptic marker Shank2 (green) and the presynaptic marker Vglut1 (red) (scale bar = 15 µm, magnification scale bar = 1 µm). Arrowheads indicate synaptic colocalization. **d** Western blot analysis and quantification of hippocampal homogenates and the P2 fraction using antibodies directed against the postsynaptic protein Shank2 and the presynaptic marker Synapsin 1/2. **e** The overall number and ultrastructure of excitatory synapses within the CA3 region. Asterisks indicate the postsynaptic density (PSD) (scale bar = 0.5 µm). **f** IHC staining and quantification of excitatory synapses in the cortex using Shank2 (green) and Vglut1 (red) (scale bar = 15 µm, magnification scale bar = 1 µm). Arrowheads indicate synaptic colocalization of the proteins. **g** IHC to detect inhibitory synapses in the hippocampus using the postsynaptic marker Gephyrin (red) and the presynaptic marker Vgat (green). Relative quantification of coimmunostained synaptic puncta (arrowheads) (scale bar = 15 µm, magnification scale bar 1 µm). **h** Western blot analysis and quantification of hippocampal homogenates and P2 fraction for Gephyrin and Vgat. *N* = 3. The error bars represent the SEMs; one-way ANOVA with Bonferroni’s post hoc comparison test was performed (*P ≤ 0.05, **P ≤ 0.005, ***P ≤ 0.0005, ****P ≤ 0.0001). **i** The trace cued and contextual fear conditioning (TCCF) test protocol. The TCCF test was performed before thoracic trauma, and an auditory and contextual memory test was performed 5 and 18 days after trauma (the results of the auditory memory test and contextual memory test were compared among each group). *N* = 8; the error bars represent the SEMs; one-way ANOVA with Bonferroni’s post hoc comparison test was performed (*P* ≤ 0.05, **P ≤ 0.005, ***P ≤ 0.0005, ****P ≤ 0.0001).

We analyzed the spine density of hippocampal pyramidal neurons and interneurons in the CA1 and CA3 regions of young mice on days 5, 10, and 18 after TxT (Fig. 1a). In pyramidal neurons, the number of dendritic spines was significantly lower (up to 50%) at 5 and 10 dpi, and there were only moderate changes in spine morphology (Supplementary Fig. 1). A general recovery of spine loss was observed after 18 dpi (Fig. 1b). In contrast, no significant changes in spine density were observed in hippocampal interneurons (Fig. 1b). Co-immunostaining with antibodies directed against the excitatory postsynaptic marker Shank2 and the presynaptic protein Vglut1 revealed that spine reduction was accompanied by a loss of synaptic contacts in the CA1 and CA3 regions at 5 and 10 dpi and that this was recovered at 18 dpi (Fig. 1c). This was observed along the whole dorsal to ventral extent of the hippocampal structure (see Supplementary Materials Section 1.3). The results were confirmed by western blot analysis in homogenates and P2 hippocampal fractions; Shank2- and Synapsin 1/2-levels were unchanged in the homogenates (Fig. 1d) but significantly downregulated in synaptic P2 fractions at 5 and 10 dpi. Consistent with these findings, the analysis of excitatory synapses in the hippocampal CA3 region by TEM demonstrated a significant decrease in the number of excitatory synapses. Interestingly, the gross morphology of the remaining synapses after trauma was unchanged, as revealed by the length, thickness, and volume (V = (length/2)^2^ × thickness × π) of the PSDs (Fig. 1e). In contrast to these findings, the number of excitatory synapses in the cortex (Fig. 1f) was unchanged, as revealed by the expression of the synaptic marker proteins Shank2 and Vglut1. Moreover, analysis of inhibitory synapses by the pre- and postsynaptic marker proteins Gephyrin and Vgat showed no significant changes in synapse number and/or protein expression in the hippocampal CA1 and CA3 regions (Fig. 1g, h). Finally, young mice were exposed to cue/contextual fear conditioning 5 and 18 days after TxT. No significant differences were observed between the experimental groups with respect to the freezing response (measured 1 min after the tone) (Fig. 1i).

### Screening for potential mechanism(s) involved in synaptic loss reveals a potential role of trauma-related alterations in hippocampal CRH and BDNF expression

**Fig. 2:**
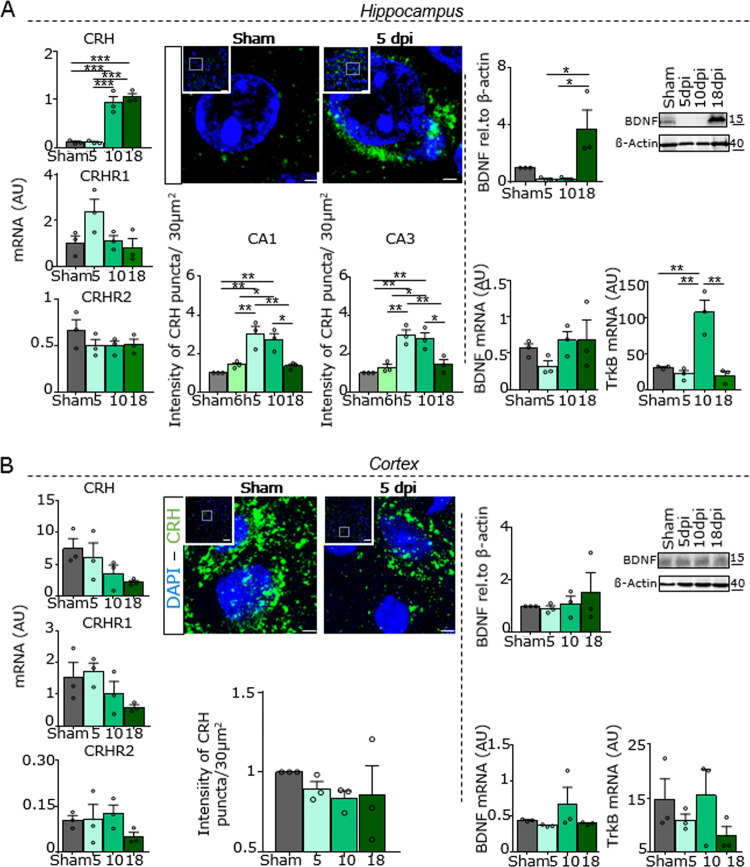
Upregulation of hippocampal CRH. **a** Expression (left) of CRH, CRHR1, and CRHR2 mRNA relative to HMBS; IHC for CRH (green) (scale bar = 15 μm, magnification scale bar = 2 μm) and quantification of CRH-positive puncta (right) in the hippocampal formation. Western blot analysis of BDNF (P2 fraction) and the relative quantification of the expression of BDNF and its receptor TrkB relative to HMBS. **b** CRH, CRHR1, and CRHR2 mRNA expression, IHC for CRH (green, scale bar = 15 μm, magnification scale bar = 2 μm), immunoblot of BDNF (P2 fraction) and the relative quantification of BDNF and TrkB expression relative to HBMS in cortical tissue. One-way ANOVA with Bonferroni’s post hoc comparison test was performed (*P ≤ 0.05, **P ≤ 0.005, ***P ≤ 0.0005, ****P ≤ 0.0001).

By using a broad screening approach to identify potential mechanisms responsible for hippocampal synaptic loss, we analyzed the potential loss of hippocampal neurons but did not find any difference between the experimental through (Supplementary Fig. 2A, G). Next, we explored the involvement of local inflammation by histological analysis of microglial (Supplementary Fig. 2B, H) and astroglial activation (Supplementary Fig. 2C, I). We observed a slight increase in microglia in the motile stage in the CA1 and CA3 regions after 10 days; however, the number and intensity of GFAP (glial fibrillary acidic protein)-positive astroglia remained unchanged. Next, we investigated the putative involvement of the complement system by using C1q as a marker of the classical complement cascade and C3b as a crucial marker of complement activation. Interestingly, C1q levels were downregulated at 5 dpi, while C3b-positive structures remained unaltered (Supplementary Fig. 2E, K). Moreover, since there is good evidence that cholinergic hypoactivity might be involved in cognitive dysfunction during delirium [31], we investigated the expression of ChAT-positive neurons in the medial septum and hippocampus (CA1 and CA3 regions). No difference in terms of the number of ChAT-positive cholinergic neurons was found between young and aged mice after TxT (Supplementary Fig. 2D, J). In addition, vital parameters after trauma did not show any significant changes, and the expression of the hypoxia-induced factor (Hif-1alpha) was not detected in the brain (data not shown). Finally, we closely analyzed the stress axis in the hippocampus first by analyzing the mRNA expression of CRH and its receptors (Fig. 2a). Here, we found that CRH mRNA was highly upregulated 10 and 18 days after trauma and CRHR1 mRNA was slightly increased at 5 dpi, while CRHR2 expression remained unchanged among the different groups. Next, we investigated the expression of CRH by evaluating the intensity of CRH-positive puncta by confocal imaging (Fig. 2a) and found that CRH expression was significantly enhanced 5 and 10 days after trauma. Moreover, we investigated the corticosterone plasma level in sham and trauma-exposed mice and detected a significant increase in the concentration (ng/ml) 6 h and 5 dpi (Supplementary Fig. 2F). Since it has been demonstrated that stress influences the expression of different neurotrophic factors [32], we screened for alterations in neurotrophin expression and found by western blot analysis that BDNF was undetectable in the hippocampal region at 5 and 10 dpi; on the other hand, there was only a slight decrease in BDNF mRNA after 5 days and a strong increase in TrkB mRNA after 10 days. In contrast, when we analyzed CRH and BDNF expression in the cortex, we did not observe any alteration in mRNA and/or protein expression (Fig. 2b).

### Unraveling the direct CRH-NF-κB-BDNF autophagy pathway that results in a rapid loss of excitatory synapses

#### CRH induces BDNF downregulation and synaptic loss in vitro

**Fig. 3:**
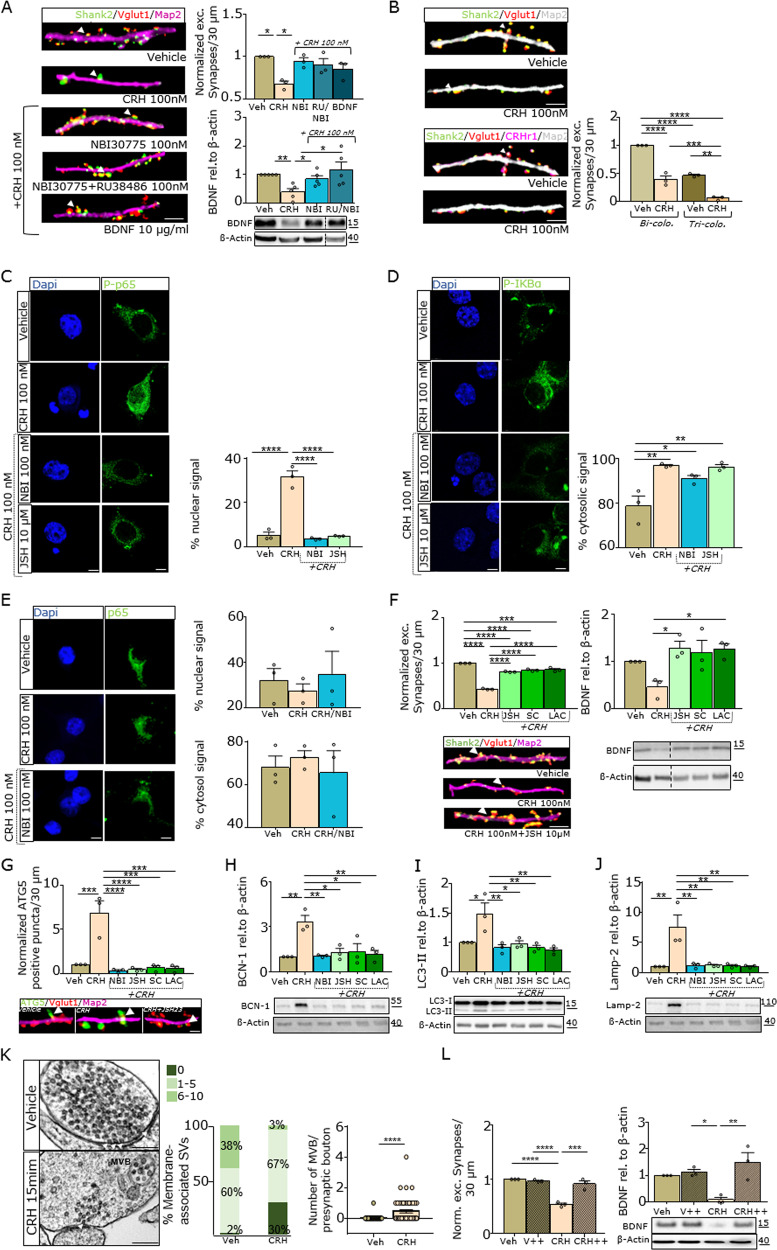
Loss of excitatory synapses in hippocampal cell culture is CRH/BDNF/NF-κB-dependent. **a** IHC for Shank2 (green), Vglut1 (red), MAP2 (magenta) (scale bar = 5 µm), western blot analysis for all different treatment regimens (CRH; the CRHR1 blocker NBI30775; the corticosterone receptor blocker RU38486; and BDNF) and the relative quantification. **b** IHC for Shank2 (green), Vglut1 (red), MAP2 (magenta) and CRHR1 (white) with colocalization and trilocalization analysis (scale bar = 5 µm). **c** IHC for phospho-NF-κB p65 (green, scale bar = 5 μm) and the relative quantification of the signal within the nuclear compartment. **d** IHC for phospho-IKB-α and the relative quantification of the signal within the cytosolic compartment. **e** IHC for and compartment analysis of p65. **f** IHC (left panel) for Shank2 (green), Vglut1 (red), MAP2 (magenta) (scale bar = 5 µm), WB of BDNF (right panel) for all different treatment regimens blocking the NF-κB pathway (JSH (the translocation blocker JSH- 23); SC (the IKK-β inhibitor SC-514); and LAC (the NF-κB activation blocker lactacystin)) and the relative quantification. **g** IHC for ATG5, immunoblot analysis of **h** BCN-1, **i** LC3, and **j** Lamp-2 and the relative quantification normalized to β-actin. **k** TEM of acquired synapses after 15 min of CRH treatment (arrows indicate membrane-associated vesicles (scale bar = 0.5 µm)) and the relative quantification of docked vesicles and multivesicular bodies (MVBs)/synapses (a two-tailed unpaired *T*-test was used). **l** Quantification of the number of excitatory synapses (left) by IHC and WB analysis of BDNF expression (right) after treatment with 100 nM CRH for 30 min and Leupeptin hemisulfate + 5 μM E64 (CRH++) compared with control treatment. *N* = 3–5; the error bars represent the SEMs; one-way ANOVA and Bonferroni’s post hoc comparison test were performed (*P ≤ 0.05, **P ≤ 0.005, ***P ≤ 0.0005, ****P ≤ 0.0001).

To study the in vivo findings in more detail, we employed a primary hippocampal/cortical culture system in which the expression of CRH and its receptors was comparable with that on day 14 after plating (Supplementary Fig. 3A). First, we tested the effect of CRH on the number of excitatory synapses 30 min after treatment with CRH (100 nM) and found a significant decrease of ~60% in the number of synapses (Fig. 3a and Supplementary Fig. 3B). Next, we analyzed whether these changes were also accompanied by a reduction in BDNF expression. To that end, we closely analyzed proBDNF, BDNF, and BDNF-mRNA levels at different time points after CRH application and found that BDNF mRNA expression was highly upregulated within the first hours, reaching steady state levels after 1 and/or 2 days of incubation. After 30 min, ProBDNF protein levels were unaltered, but the BDNF concentration was significantly downregulated, and it slowly recovered after 5 h in vitro (Supplementary Fig. 3C–E). Next, we determined the timing of CRH-induced synaptic loss and the concentration required for this effect and tested different concentrations of CRH (30 min of incubation) as well as different incubation times (with 100 nM CRH) before fixation (Supplementary Fig. 3J, G). We found a significant decrease (20%) in the number of excitatory synapses starting at a concentration of 10 nM and observed that 100 nM CRH significantly reduced the number of synaptic contacts after 5 min (loss of 13%). BDNF protein levels were clearly reduced after 30 min of CRH incubation (100 nM) (Supplementary Fig. 3G). Next, we analyzed the recovery time of hippocampal synapses by adding NBM plus B27 for 30 min or 5 h after incubation with 100 nM CRH for 30 min. Both the number of excitatory synapses and the BDNF protein expression were completely restored 5 h after medium exchange and incubation with neurobasal medium supplemented with B27, l-glutamine (2 mM, Gibco), and 100 units/ml penicillin/streptomycin (Invitrogen) (Supplementary Fig. 3H).

To further elucidate these in vitro findings, we co-applied BDNF as well as specific antagonists for CRHR1 (NBI30775) and corticosterone receptor (RU38486, mifepristone) with CRH (Fig. 3a). First, we found that the additional application of BDNF rescues the loss of synapses in comparison with CRH (100 nM) application (Fig. 3a). Moreover, NBI30775 alone and in combination with mifepristone rescued synaptic loss. Finally, we analyzed BDNF protein levels under these treatment conditions and observed unaltered BDNF expression after blockage of CRHR1 (Fig. 3a). When we investigated synaptic activity under experimental conditions by the synaptotagmin assay (Supplementary Fig. 3I) and quantified the percentage of active synapses (positive for synaptotagmin-1) in relation to the total number of excitatory synapses, we did not observe any differences among the different experimental groups, indicating that the activity of the remaining synapses after CRH application remained unchanged. Finally, we investigated the expression of CRHR1 in individual synapses and found that ~50% of excitatory synapses were CRHR1 positive. Interestingly, after CRH treatment, CRHR1-positive synapses were no longer detectable (Fig. 3b), indicating that CRHR1-positive synapses are targeted and deleted by CRH.

### CRH treatment induces autophagy activation via the NF-κB pathway in hippocampal neurons

To identify the regulatory action of CRH on BDNF levels, we employed a phospho-protein array that analyzed hippocampal neurons after treatment with CRH (100 nM) versus control. Among 1318 candidates, we found 40 phospho-proteins that were greatly upregulated or downregulated. Interestingly, the expression of NF-κB p65 itself and a group of 20 NF-κB pathway-associated proteins was upregulated after CRH treatment (Supplementary Fig. 3M). To confirm these findings, we investigated the expression and localization of phospho-NF-κB p65, NF-κB p65, and phospho-IKB-α in neurons after CRH application. Moreover, we tested the effects of the CRHR1 blocker NBI30775 and JSH23 (a specific blocker of NF-κB nuclear translocation) on NF-κB activation. We found that CRH treatment increased phospho-NF-κB p65 in the nucleus and elevated phospho-IKB-α levels in the cytosol. Such translocation was not observed after NBI30775 or JSH23 co-application (Fig. 3c, d), and the overall expression of p65 did not change after CRH administration (Fig. 3e). In addition, we confirmed the ability of CRH to induce NF-κB nuclear translocation via the canonical pathway by applying two additional compounds that blocked the NF-κB pathway at different steps of activation (SC-514 and lactacystin) (Supplementary Fig. 3L). Finally, we demonstrated that the blockage of the NF-κB pathway in combination with CRH application maintained BDNF protein expression levels and the number of excitatory synapses (Figs. 3f and 5).

Considering the rapid downregulation of BDNF protein levels, we next analyzed specific cellular degradation mechanisms that are known to be induced by the NF-κB signaling system, in particular autophagy. To that end, we tested the expression of several autophagy-related factors in vitro and found that ATG5, BCN-1, LC3, and Lamp-2 were strongly upregulated after 30 min of CRH treatment, an effect that was abolished by co-application of NF-κB and CRHR1 blockers (Fig. 3g–j). Moreover, TEM analysis of synaptic contacts revealed a high number of presynaptic terminals with MVBs and fewer membrane-associated vesicles (Fig. 3k and Supplementary Fig. 3M) after CRH treatment. Finally, we further investigated the role of lysosomal degradation by applying leupeptin hemisulfate (a reversible inhibitor of cysteine, serine, and threonine proteases) and E64 (a protease inhibitor) together with CRH and found that synapse number and BDNF levels remained unchanged when CRH treatment was combined with both inhibitors (Fig. 3l).

### The application of CRHR1 antagonists rescue posttraumatic molecular, structural, and behavioral alterations in old mice in vivo

**Fig. 4:**
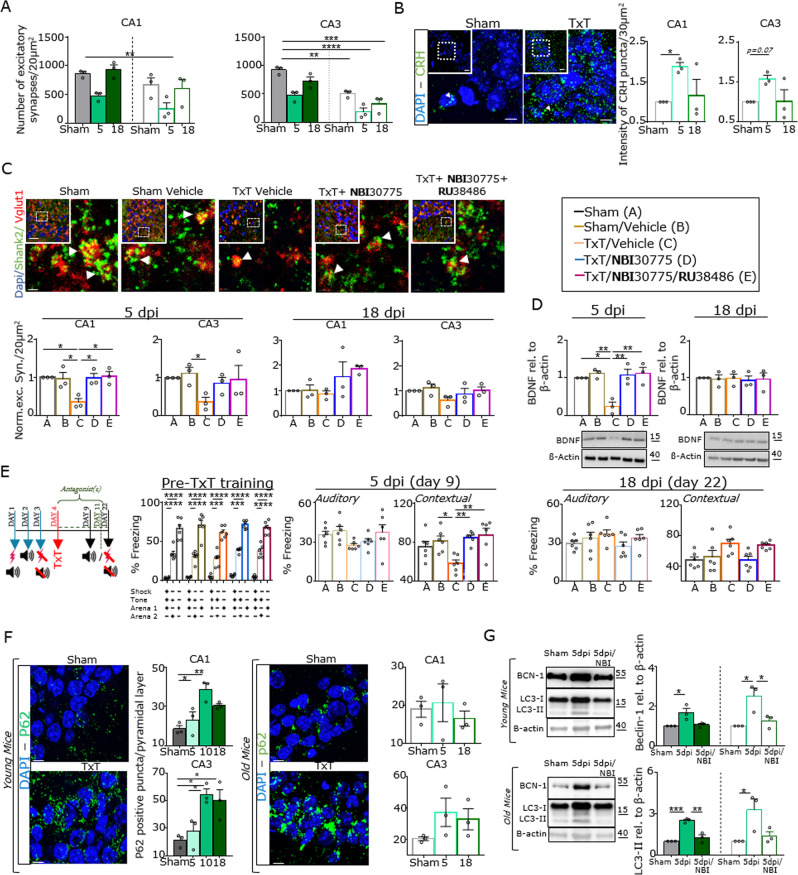
CRHR1 antagonists rescue autophagy-related hippocampal synaptic loss and memory impairment after thoracic trauma. **a** Comparison of the number of excitatory synapses in the hippocampal CA1 and CA3 regions of young and old (empty columns) mice (Shank2 and Vglut1 were used as post- and presynaptic markers). **b** CRH expression (green, scale bar = 20 μm, magnification scale bar = 5 μm) in the hippocampus of aged mice. **c** IHC for Shank2 (green) and Vglut1 (red) and colocalization analysis in the CA1 and CA3 regions of the treated groups (see internal legend) (scale bar = 15 µm, magnification scale bar = 1 µm). Arrowheads indicate synaptic colocalization. **d** Immunoblot of BDNF in the P2 hippocampal fraction and the relative quantification of the treatment groups. **e** Schematic of (left to right) the behavioral experiment, analysis of memory and learning before trauma, and analysis of auditory and contextual memory 5 and 18 days after TxT. **f** IHC for p62 in young (left) and old (right) mice in the hippocampal CA1 and CA3 regions (scale bar = 5 μm). **g** Immunoblot of Beclin-1 and LC3 in hippocampal homogenates from young and old mice 5 days after trauma with or without CRHR1 blockage. *N* = 3–6; the error bars represent the SEMs; one-way ANOVA and Bonferroni’s post hoc comparison test were always performed (*P ≤ 0.05, **P ≤ 0.005, ***P ≤ 0.0005, ****P ≤ 0.0001).

**Fig. 5:**
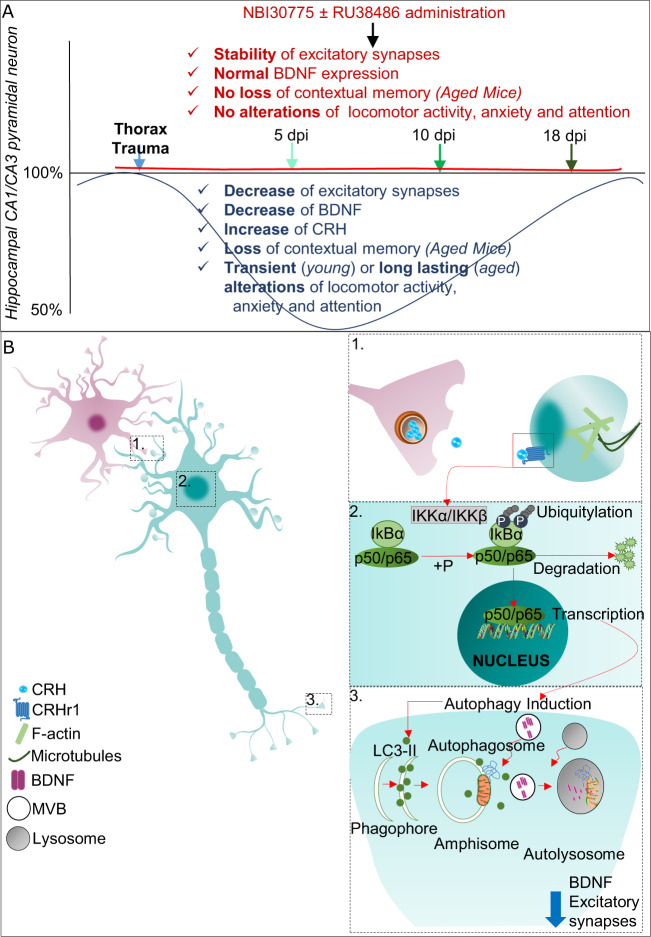
Summary of the main findings of the study. **a** Thoracic trauma (blue line) induces a selective loss of excitatory synapses in the hippocampus that is caused by CRH expression, leading to local degradation of BDNF. The physiological recovery of these parameters after TxT in mice is observed approximately at day 18 dpi. The application of CRH receptor 1 with or without the co-application of corticosterone receptor antagonists (red line) stabilizes hippocampal BDNF expression. Thereby, the morphological and behavioral alterations are rescued. **b** A thumbnail sketch of the proposed underlying cellular and molecular mechanisms. Peripheral trauma (here, TxT) induces a pronounced elevation of the hippocampal release of CRH, which activates the canonical NF-κB pathway via synaptic CRHR1s. This activation induces the autophagy machinery, leading to the local degradation of BDNF and eventually to the loss of excitatory synapses in the hippocampus.

In light of age-dependent alterations in synaptic plasticity [33] and the enhanced susceptibility of elderly patients to posttraumatic neuropsychiatric symptoms, we finally investigated the effects of trauma in aged mice compared with younger animals. Comparing the absolute number of hippocampal excitatory synapses between young and older mice showed that old animals had fewer excitatory synapses before trauma. In particular, in the CA3 region, this difference reached ~50%. After the induction of TxT in the older mice, a 50% reduction in the number of synapses was observed (5 dpi), and this reduction slowly recovered after 18 dpi (Figs. 4a and 5). These data also indicated that after TxT, old mice had only ~20–25% of the number of hippocampal excitatory synapses that young WT mice had. The concomitant upregulation of hippocampal CRH was also observed to a similar extent in old animals (Fig. 4b).

Based on these results and the in vitro results, we next designed an experimental trial to evaluate the therapeutic potential of CRHR1 antagonists (and mifepristone) after trauma in old (Fig. 4) and young animals in vivo (Supplementary Fig. 4). We did not detect a reduction in the number of excitatory synapses in trauma-exposed animals that received the antagonists compared with trauma-exposed animals (C), which exhibited 50–60% synaptic loss at 5 dpi. In the trauma-exposed group at 18 dpi, synapses naturally recovered so that no differences between the treatment groups were observable when the CA1 and CA3 regions were analyzed (Fig. 4c and Supplementary Fig. 4A). As proof of principle, we also analyzed BDNF protein levels in the hippocampus under these experimental conditions and observed unchanged BDNF levels (Fig. 4d and Supplementary Fig. 4C). Finally, mice were exposed to cue/contextual fear conditioning before trauma and on 5 days and 18 days after trauma (Fig. 4e and Supplementary Fig. 4B). All groups showed an increase in freezing responses during the auditory/contextual memory test in the pre-TxT period. In older mice exposed to trauma and treated with NBI30775 alone or in combination with mifepristone, lesions of the hippocampus appeared to interfere with the acquisition of contextual freezing responses but not cue-elicited freezing responses 5 days after trauma (Fig. 4e and Supplementary Fig. 4B). To further validate our model and to make it comparable with other published delirium models, we expanded the phenotypical characterization of all experimental groups by performing a battery of behavioral tests (the open field test, Y maze, and elevated plus-maze) [34–39] on young (Supplementary Fig. 5A–C) and aged mice (Supplementary Fig. 5D–F) 24 h prior to TxT and 4 h, 24 h, 5 days and 18 days after trauma. We found a strong impairment of locomotor activity, cognitive performance and anxiety behavior in young and old trauma-exposed mice at 4 h post injury. These alterations quickly recovered in young animals but were still evident in older mice at 24 hpi and 5 days after trauma. Interestingly, the administration of NBI30775 alone or in combination with mifepristone reversed nearly all the observed behavioral alterations after trauma in young and aged animals.

Finally, we analyzed the involvement of autophagy in posttraumatic synaptic loss under in vivo conditions and used antibodies directed against p62, BCN-1, and LC3 (Fig. 4f, g). Regarding p62, we found an increase in p62-positive puncta in the hippocampi of younger mice, particularly after 10 days. In older mice, we observed the same trend at 5 dpi. The hippocampal protein expression of BCN-1 and LC3 after trauma was found to be significantly upregulated in younger and aged mice at 5 dpi; this effect was abrogated by NBI30775 administration (Fig. 4g).

## Discussion

Physical trauma or complicated, long-lasting surgical operations are closely associated with neuropsychiatric syndromes that are subsumed as acute physical stress response/disorder or delirium [40, 41]. In industrial countries, delirium affects ~15% of all inpatients [42, 43] and is highly correlated with higher mortality and extended hospital stays [44]. Despite the great efforts of scientists and clinicians to unravel the pathophysiology of delirium and to develop effective treatment options, this acute and mostly transient neuropsychiatric disorder is still far from being completely understood. This is also because the construct, face and predictive validity of animal models of delirium is very limited due to the multiple causes and broad spectrum and variety of symptoms. In one of the first rodent delirium models, the muscarinic receptor antagonist atropine was administered and led to acute cognitive deficits in a blind alley maze and EEG slowing reminiscent of delirium [45]. Animals with selective lesions of the basal forebrain cholinergic system have been shown to be susceptible to acute cognitive dysfunction that is reversible upon inflammatory resolution [31]. In fact, more recently, several delirium models based on the induction of an inflammatory response were introduced. Delirium-like behavioral alterations are observed after the application of high doses of lipopolysaccharide (LPS) or cecal ligation and puncture leading to polymicrobial sepsis [46]. For example, Chen et al. [47] showed that compared with younger mice, old mice exhibit an increased inflammatory response in the hippocampus after LPS challenge, and it was found that hippocampal processing is more easily disrupted in old animals than in younger ones. In line with these findings, in ME7 animals (a mouse model of prion disease), the application of LPS induces acute and transient working memory deficits, and ME7 animals show heightened and prolonged transcription of inflammatory mediators in the CNS [48].

In this study, we wanted to mimic postoperative [39] and posttraumatic cognitive dysfunction without primarily triggering inflammatory responses. To that end, we induced peripheral trauma (TxT) to analyze the molecular, cellular, and behavioral features of delirium. Interestingly, in this new rodent model, we observed a broad spectrum of behavioral deficits (especially in aged animals) reminiscent of delirium. We found that TxT has a significant impact on the homeostasis of hippocampal excitatory synapses. In aged animals, the transient 50% synapse reduction in the hippocampus resulted in an impairment of memory formation (Fig. 5). The loss of synapses may have been attributable to the autophagic degradation of BDNF that induced by local hippocampal release of CRH through the CRHR1/NF-κB pathway. Blocking CRHR1 rescued delirium-like symptoms in vivo. Our data suggest that the pathophysiological basis of the rapid decline in cognitive function might indeed be explained by synaptic loss and subsequent functional impairment of local circuitries within the hippocampal formation [19].

To exclude other putative mechanisms/factors that are known to regulate synapse number, we closely analyzed neuronal loss, hypoxia and complement cascade involvement [49, 50] as well as severe signs of neuroinflammation. As mentioned before, these mechanisms have also been proposed to cause delirium (mainly based on clinical data sets [4]). We especially focused on microglial and astroglial activation [51–53] but we were unable to detect specific signs of inflammatory responses after TxT.

Interestingly, the synaptic alterations that were found were brain region- and neuron/synapse-specific, indicating a selective vulnerability of the hippocampus to peripheral trauma. This regional specificity was most likely due to the local expression of CRH in hippocampal interneurons, as in vitro data indicated that cortical neurons were CRH-responsive in principle. Moreover, our experiments on primary hippocampal neurons indicated that synapses involving the dendritic trees of pyramidal neurons expressing CRHR1 were specifically deleted. Of note, the remaining excitatory synapses showed no morphological differences compared with synapses under sham conditions and showed no differences in functional properties in the synaptotagmin assay.

It is well known that stress influences memory by modulating the integrity and plasticity of synapses, which are fundamental for memory processes [54–57]. Here, we propose a novel mechanistic concept of the development of delirium based on the idea of hippocampal stress response [12, 58–61] triggered by an elevation of posttraumatic interneuronal CRH production and release [62].

There are important published data on the molecular mechanisms by which a reduction in the number of spines and synapses is induced by CRH release. The authors proposed that CRH-CRHR1-mediated activation of a Rho-GTPase induces actin depolymerization and subsequent spine loss [61, 63]. Based on these data, we screened for additional potential mediators of the action of CRH on pyramidal neurons and found that BDNF [15], a prominent signaling molecule that influences synaptogenesis and spine formation [64], neuronal survival [65], LTP, neuronal excitability [66], and adult hippocampal neurogenesis [67], was almost entirely depleted in the hippocampus after TxT.

Eventually, we identified a novel pathway by which CRH exerts its effect on BDNF and found that the NF-κB signaling cascade is essentially involved. To our knowledge, this is the first study that identifies a CRH-CRHR1-NF-κB interaction in primary neurons (Fig. 5). However, it has already been shown that CRH specifically activates the NF-κB pathway via CRHR1 in thymocytes [68, 69]. Furthermore, it has been shown that the activated NF-κB pathway can induce autophagy by upregulating BCN-1 [70]. Indeed, we observed in our experimental setup that BCN-1, LC3, and Lamp-2 were significantly upregulated by elevated CRH levels in neurons (in vitro and in vivo) and that signs of autophagy induction, especially in the presynaptic compartment, appeared. Interestingly, recent evidence of fine-tuned protein homeostasis regulated by autophagy at synapses, especially in the presynaptic compartment, was reported [71–73]. We found that CRHR1 activation was correlated with the rapid downregulation of (presynaptic) BDNF and that BDNF degradation was rescued by CRHR1 blockage as well as NF-κB pathway inhibition. Finally, we tested the hypothesis that elevated CRH levels induce rapid lysosomal/autophagic BDNF degradation in hippocampal neurons [74] and applied proteosomal inhibitors. We thereby stabilized BDNF levels as well as synapse integrity in vitro, supporting the novel concept of a direct regulatory link between CRH and BDNF. The loss of synapses is therefore explained by the local disruption of a proposed BDNF autocrine feed-forward loop [75].

As already mentioned before, age is one of the strongest predictors of the development of delirium after trauma, and aged-related neurodegeneration has been discussed as an additional predisposing factor (“second hit”) for the development of delirium [48, 76]. When we compared young and old animals, we did not observe significant differences in the stress reaction in the brain per se; however, the number of hippocampal synapses was already reduced to ~50% before the traumatic event in the aged mice. This might explain why behavioral deficits after trauma are more severe in elderly patients; it appears much more likely that the total number of synaptic connections falls below a certain threshold, guaranteeing hippocampal functionality. By applying CRHR1 antagonists, however, we successfully rescued synapse numbers and reversed all accompanying histological, molecular and behavioral changes observed after trauma in young and aged animals. The results are also in very good accordance with data obtained from CRHR1 KO mice, which have fewer hippocampal spines than control animals and do not exhibit downregulated spines/synapses in response to early life stress [77].

Based on our results, a posttraumatic delirium-like status might be prevented by inhibiting the CRH-CRHR1-NF-κB-BDNF pathway. Several antagonists of the CRH receptor, the best known of which are the selective CRHR1 antagonist Antalarmin and a newer drug pexacerfont, have been developed and are widely used in research. A recent human trial found that pexacerfont was not effective in alleviating the symptoms of general anxiety disorder [78], although additional research is still needed. In monkeys, Antalarmin has been shown to be successful in lowering the stress-induced CRF rise in CSF, suppressing anxiety-associated behaviors, and increased exploratory behavior in stressful situations, but human trials are necessary to test the clinical efficacy of Antalarmin [79–82]. In our study, we applied the CRHR1 antagonist NBI30775, which has already been used in clinical trials for depression/anxiety and has shown good efficacy and safety [83, 84]. Moreover, there are trials using various compounds that block the NF-κB pathway, especially for the treatment of cancer, that might also benefit after trauma events [85].

In summary, our study provides a novel model of acute physical stress responses and delirium as well as molecular insights into posttraumatic (especially synaptic) changes in the brain that are initiated by the elevation of hippocampal CRH levels (Fig. 5). The therapeutic interruption of this novel trauma-induced signaling cascade might therefore prevent synaptic loss and eventually the occurrence of delirium and reduce its severity.

## Supplementary information

SupplMaterial

SuppFig1

SuppFig2

SuppFig3

SuppFig4

SuppFig5

## Data Availability

The authors confirm that the data supporting the findings of this study are available within the article [and/or] its supplementary materials.
