# Supplementary material for: A CRHR1 antagonist prevents synaptic loss and memory deficits in a trauma-induced delirium-like syndrome
Source: Mol Psychiatry. 2020 Feb 12;26(8):3778–94. doi: 10.1038/s41380-020-0659-y (PMC8550963; doi:10.1038/s41380-020-0659-y)
Supplement: Supplementary file 1 — SupplMaterial [file 41380_2020_659_MOESM1_ESM.docx]

**Supplementary Materials:**

**1.1 Study Design**

The research objective of our study was to determine the structural and molecular mechanisms underlying (peripheral) trauma induced pathophysiological alterations of the CNS leading to neuropsychiatric syndromes like delirium. As a model, we used the well-established thorax trauma (TxT) model in mice. TxT is induced by a blast wave. All mice were assigned randomly to each experimental group. For the *in vitro* experiments, primary hippocampal neurons from embryonic rats and mice were cultured. No data were excluded. The statistical analysis paragraph includes the details regarding the replicates used to generate the different data sets.

**1.2 Spine morphology**

**Spine morphology was identified manually using ImageJ software according to the classification from (**[***1***](#_ENREF_1)**)**.

**1.3 Immunostaining Analysis**

*Analysis of colocalized synapse dorsal/ventral hippocampus*

For orientation analysis synapses were investigated on the overall extension of the hippocampus and similar reduction was found. For statistical evaluation colocalized synapses were identified and counted at two predefined areas moving rostro-caudally (area1: -1.35 mm -1.95 mm, area2: -2.78 mm -3-38 mm). Three immunohistochemical labeled sections per area for each mouse (n=3) were acquired by using Leica SPE confocal microscope with a 40x/63x objectives. Images were analyzed using Bitplane Imaris Software: three random areas (20μm^2^) were taken for every picture with only the DAPI channel active, then an intensity threshold was set and puncta per channel were visualized. Finally, the colocalization tool was applied obtaining the sum of the colocalized synapse in the three area previously selected.

*Analysis of Intensity signals*

Fiji ImageJ Software was used for all the intensity measurements (CRH, IBA1, GFAP, phospho NF-κB p65, NF-κB p65 and phospho IKB-α) of single cells or three regions of interest/image. From the Analyze menu from “set measurements”, area integrated intensity and mean grey value were selected. Moreover, a region with no fluorescence was selected as background.

*Analysis of microglial activation: visual characterization*

Iba1 positive cells were counted and morphologically characterized based on the activation stage described by Kreutzberg ([*2*](#_ENREF_2)). The microglial morphology was classified as either resting (small round cell body with thin multiple processes), withdrawal (elongated cell body with thicker cellular processes) or motile (round or amoeboid cell body with almost no processes) (**Table 1**).

The number of activated microglia expressed as a percentage of the total number of microglia was used as a measure for microglial activation.

**
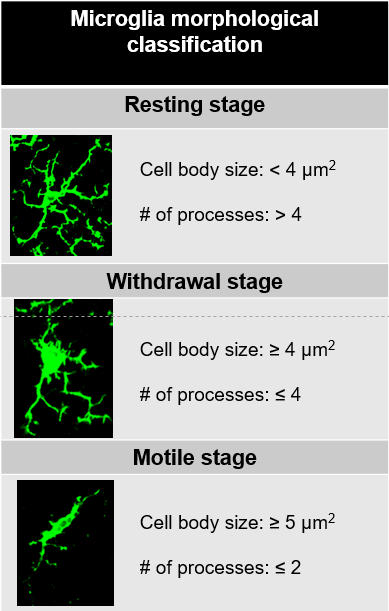
**

**Table 1. Morphological characterization of IBA1+ cells**

**1.4 Behavioral assessment**

Locomotor activity was assessed in an open field. Mice were placed in a box (50 x 50 x 40 cm), and the number squares crossed were recorded over 2 minutes ([*3*](#_ENREF_3)). In order to assess short memory in mice the Y- maze test (40 x 7 x 15 cm) was performed for 5 minutes: spontaneous alternation, a measure of spatial working memory, can be assessed by allowing mice to explore all three arms of the maze and is driven by an innate curiosity of rodents to explore previously unvisited areas. A mouse with intact working memory, will remember the arms previously visited and show a tendency to enter a less recently visited arm ([*4*](#_ENREF_4)). To assess anxiety-related behavior, the elevated plus maze (30 x 5 x 15 cm) was used: briefly, mice are placed at the junction of the four arms of the maze, facing an open arm, and entries/duration/tracklength in each arm are recorded by a video-tracking for 5 min ([*5*](#_ENREF_5)). For all these behavioral paradigms, the BioObserve Viewer^3^ Software was used.

**1.5 Corticosterone measurement**

In brief, animals were killed with an overdose of sevoflurane and exsanguinated by cardiac puncture. Plasma samples were collected in heparine monovette (SARSTEDT) and immediately centrifuge at 2000g 4 °C for 10 min. Then, the surnatant was processed following the protocol and the precautions suggested by the Corticosterone Elisa Kit (IBL International, RE52211).

**Supplementary Figures Legends**

**Fig.S1 Analysis of spine morphology after TxT**. Golgi staining in CA1 (**A**) and CA3 (**B**) region. N= 3, error bars represent mean + SEM, *one-way ANOVA correct for multiple comparison and Bonferroni’s Comparison test as post hoc test* was always performed. (*P=0.05, **P=0.005, ***P=0.0005, ****P≤0.0001).

**Fig. S2 Additional parameters determined after TxT (A)** IHC stainings for the neuronal marker NeuN (green) and Dapi (blue) with relative quantification of positive nuclei for NeuN in CA1 and CA3 (scale bar 15 μm). (B) IHC for the microglial marker Iba1 (green) and Dapi (blue) (scale bar 7 μm) with relative classification of microglia cell activation state. R: resting W: withdrawal M: motile (C) IHC for the astrocyte marker GFAP (red) and Dapi (blue) (scale bar 7 μm) with relative quantification of intensity and positive cells in the CA1 and CA3 region. (D-J) IHC staining for the ChAT positive neurons (green) and Dapi (blue) in medial septum with relative quantification of positive cells in medial septum, CA1 and CA3 (scale bar 50 μm). € Immunohistochemistry for C1q (green, scale bar 15 μm) in CA1 and CA3 with relative quantification of C1q positive puncta; immunoblot of C3b/iC3b/C3c (P2 fraction) with quantification relative to β-actin. (F) ELISA for quantification of corticosterone concentration in plasma. (G) IHC for NeuN in CA1 and CA3 of aged mice with quantification of positive cells (green, scal bar 5 µm). (H) IHC for Iba1 and analysis of three different activation state in older mice (green, scal bar 10 µm). (I) IHC for GFAP in hippocampus with relative quantification of number of positive cells and intensity of GFAP signal in older mice (green, scal bar 10 µm). (K) IHC for C1q (green, scal bar 5 µm) in CA1 and CA3 with relative intensity analysis. N=3-5, error bars represent mean+SEM. *One-way ANOVA and Bonferroni’s Comparison test as post hoc test* was always performed (*P=0.05, **P=0.005, ***P=0.0005, ****P≤0.0001).

**Fig. S3** **Loss of excitatory synapses in hippocampal cell culture is CRH/BDNF/NF-κB dependent** (A) Analysis of CRH, CRHR1 and CRHR2 mRNA expression at DIV 7, 14 and 21 in hippocampal (HN) (left) and cortical (CN) (right) neurons. (B) Quantification of excitatory synapses number after IHC (colocalization of Shank2/Vglut1/30μm of dendrites) of HN (left) and CN (right) at DIV14 after 30min treatment. (C) Immunoblot of pro-BDNF relative to β-actin. (D-E) quantification of BDNF mRNA and peptide level after different treatment time. (F) Quantification of synaptotagmin-1 positive excitatory synapses after 30min of CRH 1nM treatment (tri-colocalization among Shank2/Vglut1/Synaptotagmin 1/30μm of dendrites). (G) WB analysis of BDNF expression (top) and quantification of excitatory synapses number (bottom) after IHC, after different CRH 100nM incubation time (*two tailed unpaired t-test* was always performed, all the conditions were compared to the Vehicle=1, WB for BDNF was analyzed by *One-way ANOVA with Bonferroni’s Comparison test as post hoc test).* (H) WB analysis of BDNF expression (top) and quantification of excitatory synapses number (bottom), after treatment with CRH 100 nM/30 min followed by NBM plus B27 for 30 min and 5 hours. (I) IHC for Synaptotagmin-1(cyan), Vglut1(red), Shank2(green) and MAP2(magenta) (scale bar 5µm) for all different treatment regimen with relative quantification. (L) Analysis of excitatory synapses number after treatment with CRH 1-3-6-10nM. (M) Screening of phosphoprotein represented as percentage of difference among vehicle and CRH 100 NM. (N) IHC for phospho NF-κB p65 after CRH treatment ± SC-514 and Lactacystin. (O) Overview of the synaptic contact magnified in Fig.3M (right) and other example of differences among vehicle and CRH in term of presence of MVB and decrease of docked vesiscles.  *One-way ANOVA followed by Bonferroni’s Comparison test or Dunnett’s as post hoc test* was performed (*P=0.05, **P=0.005, ***P=0.0005, ****P≤0.0001).

**Fig. S4** **Applications of CRH and corticosterone antagonists rescue posttraumatic hippocampal alterations *in vivo*** (A) IHC staining of hippocampal sections using Shank2- (postsynaptic, green) and Vglut1- (presynaptic red) specific antibodies (scale bar 15 µm, high magnification scale bar 1 µm). Quantification of synapse number (colocalized puncta) under all experimental conditions at 5 dpi and 18 dpi. (B) Schematic representation of behavior analysis, TxT and drug/s administration. Increase of freezing response during the tone/context memory shown on the pre-TxT training (n=6/group); no significant difference of freezing response after trauma between the groups. (C) Immunoblot for BDNF after CRH ± antagonist(s) with relative quantification. N= 3-6, error bars represent mean + SEM*, One-way ANOVA with Bonferroni’s Comparison test as post hoc test was* always performed (*P=0.05, **P=0.005, ***P=0.0005, ****P≤0.0001).

**Fig. S5** **Validation of delirium-model thorax trauma-induced via behavioural paradigm** (A) Analysis of squares crossed in the open field, (B) number on Spontaneous Alternations Performance (SAP) in the Y-maze and (C) time spent in the Open Arms (OA) vs tracklength in the Closed Arms and in young mice and old mice (D, E and F respectively) 24 hours prior thorax trauma, 4 hours, 24 hours and 5 days after trauma.N=6, error bars represent mean + SEM*, One-way ANOVA with Bonferroni’s Comparison test as post hoc test was* always performed (*P=0.05, **P=0.005, ***P=0.0005, ****P≤0.0001).

**References**

1. Hering H, Sheng M, Dendritic spines: structure, dynamics and regulation. *Nature reviews. Neuroscience* 2001*;* **2**, 880-888.

2. Kreutzberg GW, Microglia: a sensor for pathological events in the CNS. *Trends in neurosciences* 1996*;* **19**, 312-318.

3. Hennessy E, Gormley S, Lopez-Rodriguez AB, Murray C, Murray C, Cunningham C, Systemic TNF-alpha produces acute cognitive dysfunction and exaggerated sickness behavior when superimposed upon progressive neurodegeneration. *Brain, behavior, and immunity* 2017*;* **59**, 233-244.

4. Kraeuter AK, Guest PC, Sarnyai Z, The Y-Maze for Assessment of Spatial Working and Reference Memory in Mice. *Methods in molecular biology* 2019*;* **1916**, 105-111.

5. Walf AA, Frye CA, The use of the elevated plus maze as an assay of anxiety-related behavior in rodents. *Nature protocols* 2007*;* **2**, 322-328.
