# Supplementary figures and images for: A CRHR1 antagonist prevents synaptic loss and memory deficits in a trauma-induced delirium-like syndrome
Source: Mol Psychiatry. 2020 Feb 12;26(8):3778–94. doi: 10.1038/s41380-020-0659-y (PMC8550963; doi:10.1038/s41380-020-0659-y)

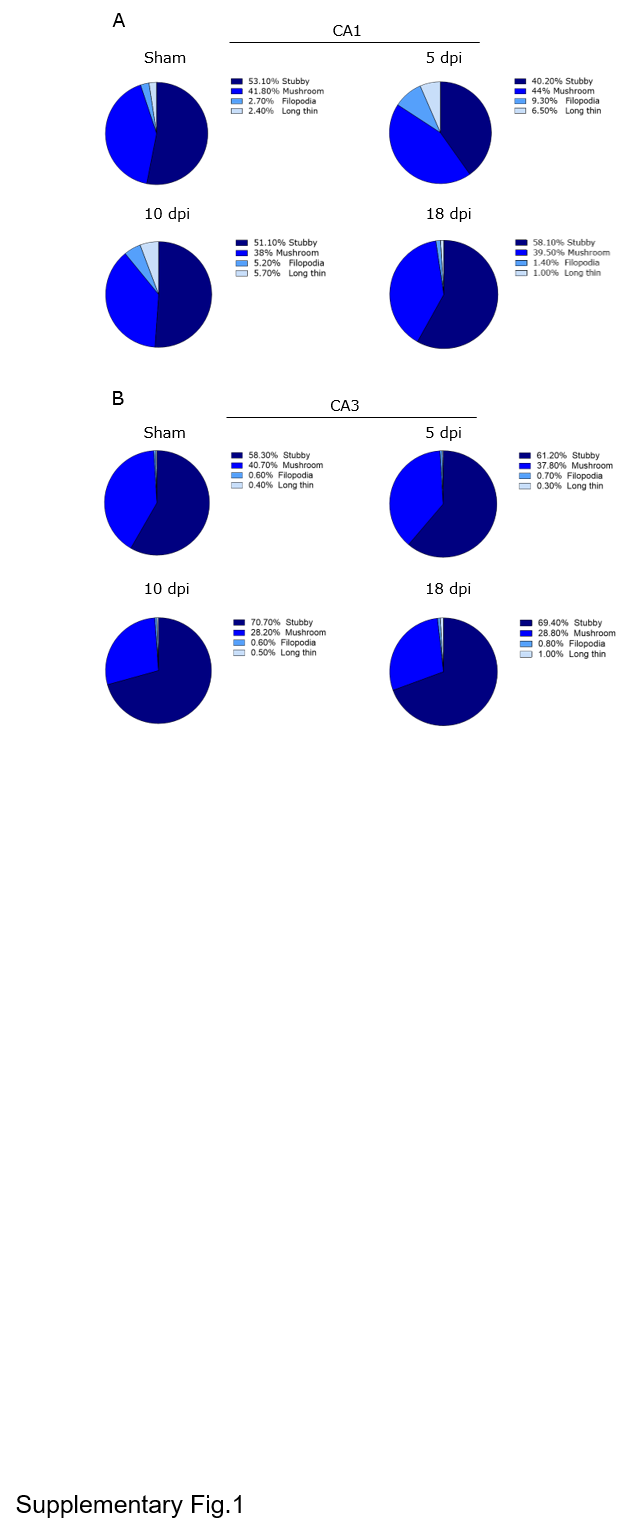

Supplement: Supplementary file 2 — SuppFig1 [file 41380_2020_659_MOESM2_ESM.tif]

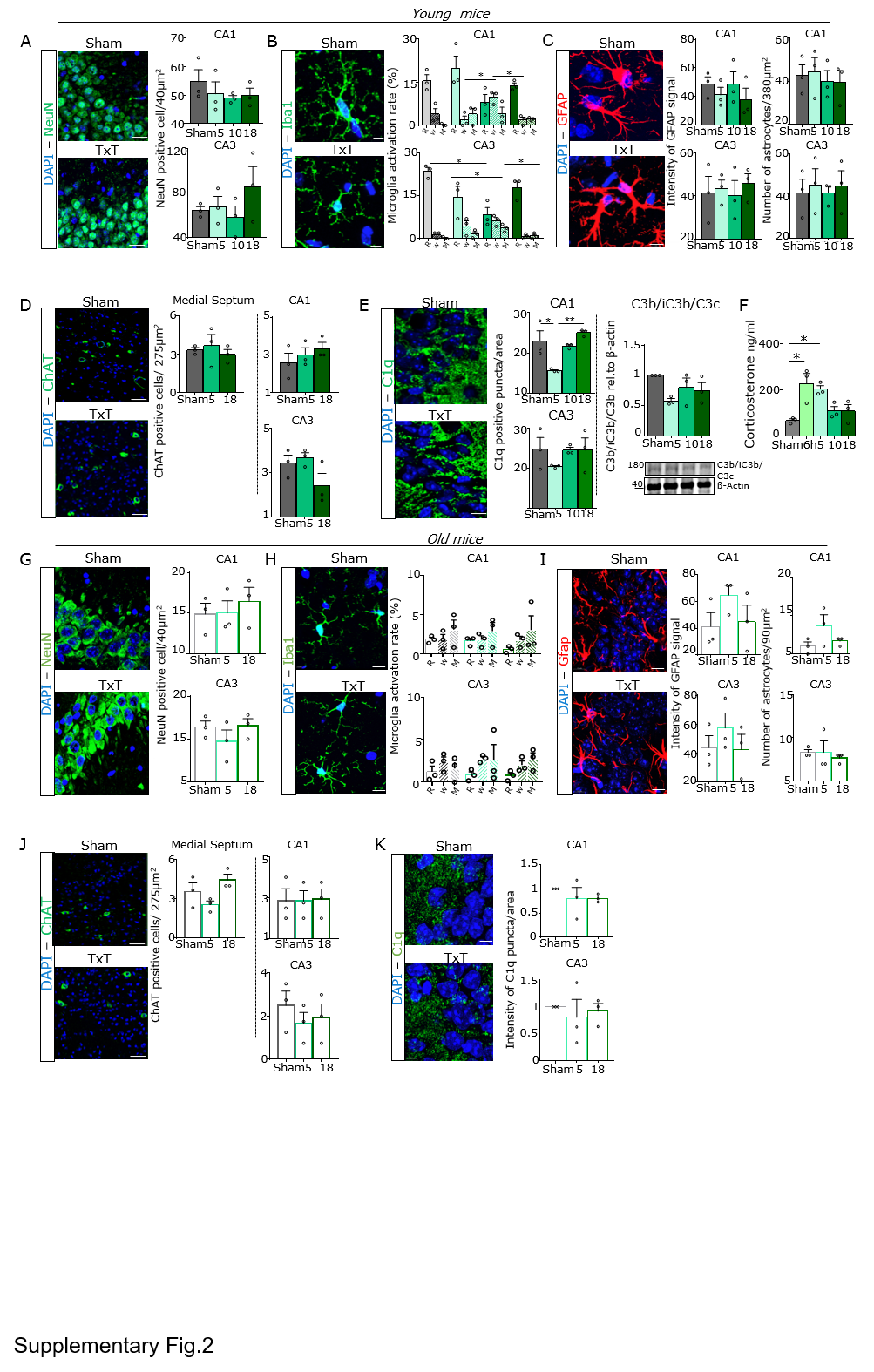

Supplement: Supplementary file 3 — SuppFig2 [file 41380_2020_659_MOESM3_ESM.tif]

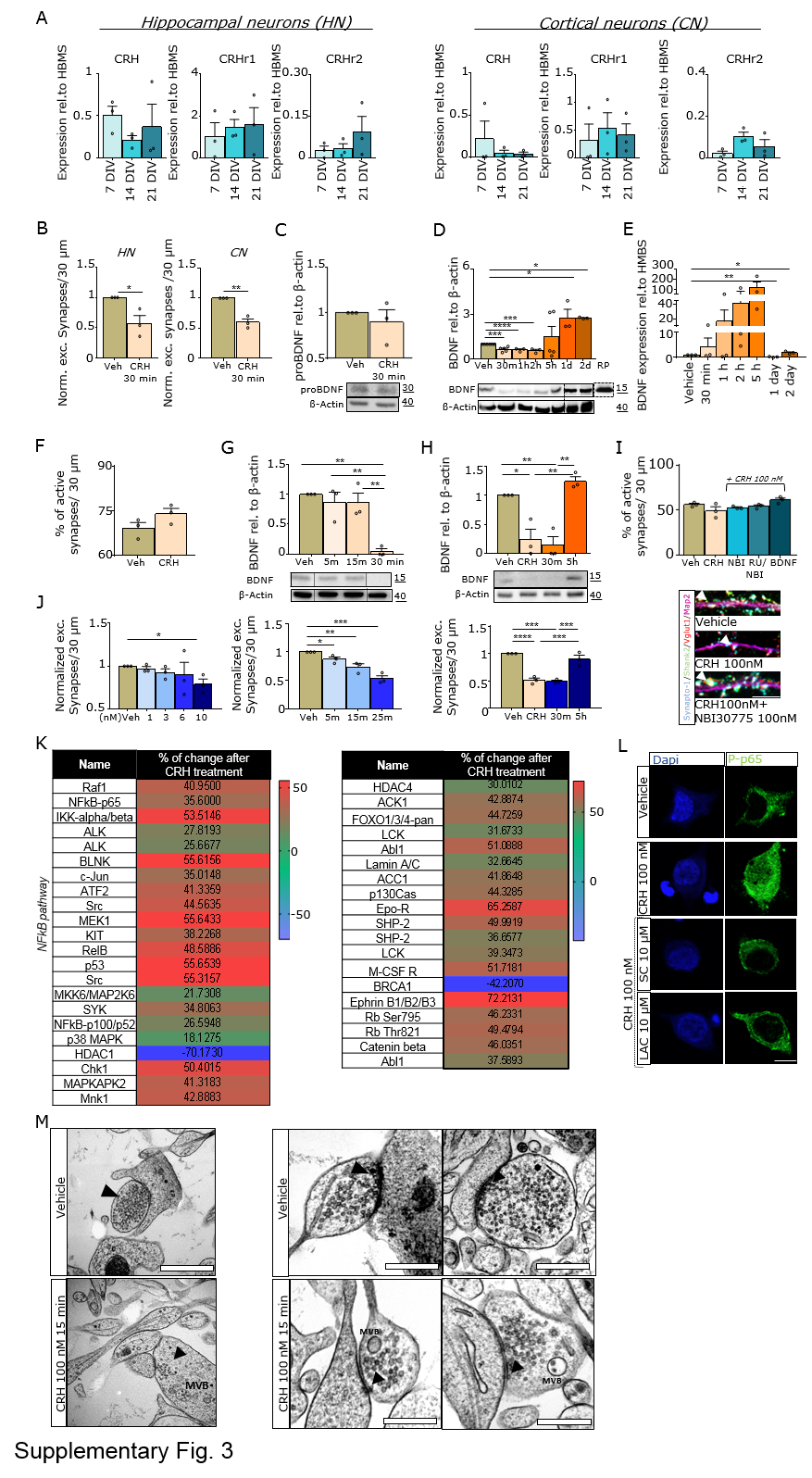

Supplement: Supplementary file 4 — SuppFig3 [file 41380_2020_659_MOESM4_ESM.tif]

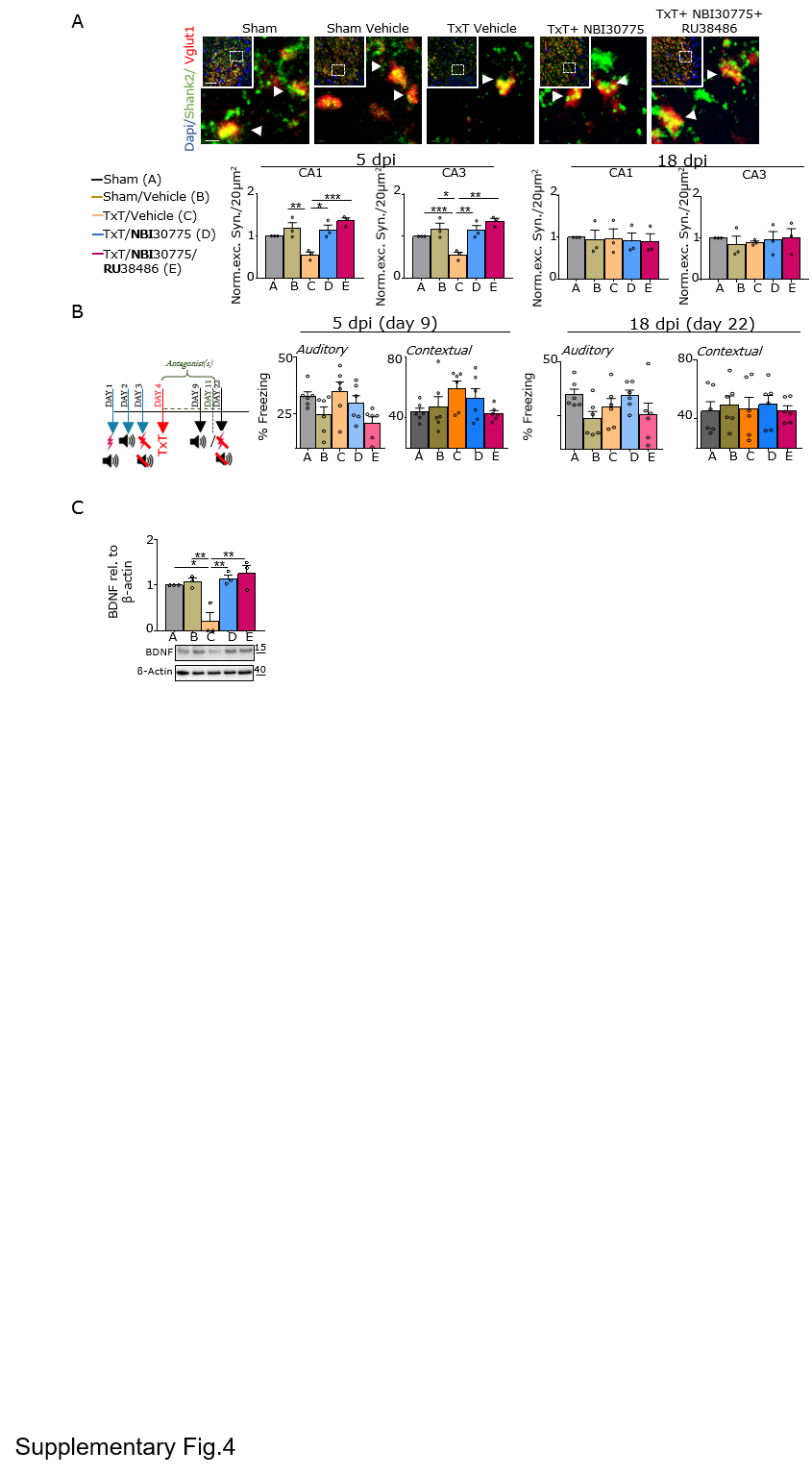

Supplement: Supplementary file 5 — SuppFig4 [file 41380_2020_659_MOESM5_ESM.tif]

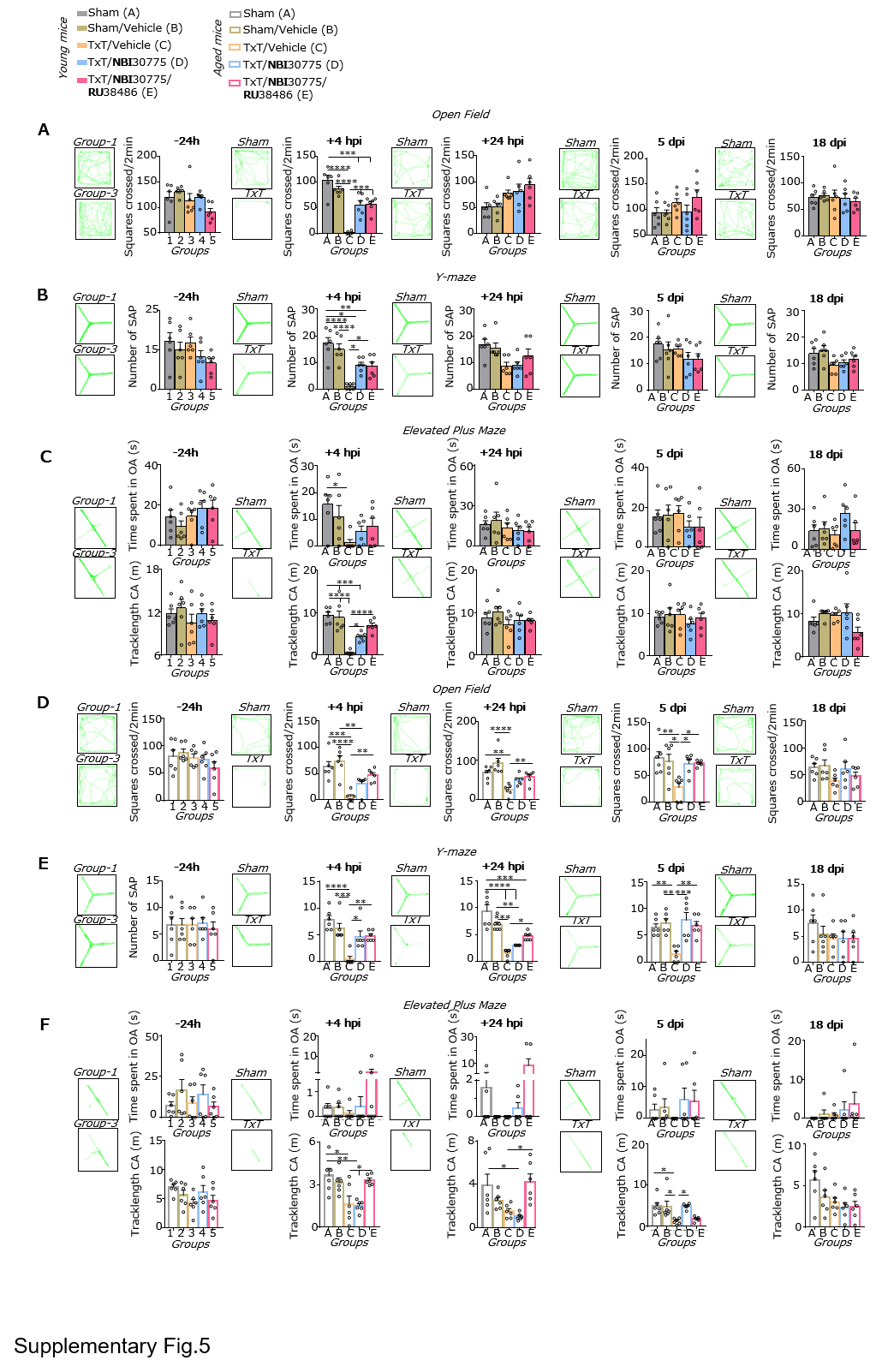

Supplement: Supplementary file 6 — SuppFig5 [file 41380_2020_659_MOESM6_ESM.tif]
